# Clinical and Epidemiological Characterization of Laboratory-Confirmed Autochthonous Cases of Zika Virus Disease in Mexico

**DOI:** 10.1371/currents.outbreaks.a2fe1b3d6d71e24ad2b5afe982824053

**Published:** 2016-04-15

**Authors:** Maria Eugenia Jimenez Corona, Ana Lucía De la Garza Barroso, Jose Cruz Rodriguez Martínez, Norma Irene Luna Guzmán, Cuitláhuac Ruiz Matus, José Alberto Díaz Quiñonez, Irma Lopez Martinez, Pablo A. Kuri Morales

**Affiliations:** General Directorate of Epidemiology, Ministry of Health, Mexico City, Mexico; Dirección General Adjunta de Epidemiología, Dirección General de Epidemiología, Secretaría de Salud, Mexico City, Mexico; Dirección de Vigilancia de Enfermedades Transmisibles, Dirección General de Epidemiología, Secretaría de Salud, Mexico City, Mexico.; Dirección General de Epidemiología, Secretaría de Salud, Mexico City, Mexico; Dirección General de Epidemiología, Secretaría de Salud, Mexico City, Mexico; Facultad de Medicina, Universidad Nacional Autónoma de México, Mexico City, Mexico; Instituto de Diagnóstico y Referencia Epidemiológicos (InDRE) "Dr. Manuel Martínez Báez", Mexico City, Mexico; Instituto de Diagnóstico y Referencia Epidemiológicos (InDRE) "Dr. Manuel Martínez Báez", Mexico City, Mexico; Subsecretaría de Prevención y Promoción de la Salud, Secretaria de Salud, Mexico City, Mexico

**Keywords:** arbovirus, Epidemiology, Surveillance, Zika

## Abstract

Introduction: Since 2014, autochthonous circulation of Zika virus (ZIKV) in the Americas was detected (Easter Island, Chile). In May 2015, Brazil confirmed autochthonous ­­transmission and in October of that year Colombia reported their first  cases. Now more than 52 countries have reported cases, including Mexico. To deal with this contingency in Mexico, several surveillance systems, in addition to systems for vector-borne diseases were strengthened with the participation of all health institutions. Also, the Ministry of Health defined an Action Plan against ZIKV for the whole country.

Methods: We analyzed 93 autochthonous cases of ZIKV disease identified by Epidemiological Surveillance System for Zika Virus in Mexico. All autochthonous cases confirmed by laboratory since November 25, 2015 to February 19, 2016 were included. A description of clinical and epidemiological characteristics of 93 cases of ZIKV disease are presenting and, we describe the Action Plan against this public health emergency.

Results: The distribution of cases by sex was 61 men and 32 women; mean age was 35 years old (S.D. 15, range 6-90). The main clinical features in the 93 cases were fever (96.6%), rash (93.3%), non-purulent conjunctivitis (88.8%), headache (85.4%), and myalgia (84.3%). No deaths were reported.

Conclusion: The ZIKV epidemic poses new challenges to public health systems. The information provided for basic, clinical, and epidemiological research, in addition to the data derived from epidemiological surveillance is essential. However, there are still many unanswered questions regarding mechanisms of transmission, complications, and impact of this virus.

## INTRODUCTION

Zika virus (ZIKV) is a flavivirus related to yellow fever, dengue, West Nile viruses, and Japanese encephalitis viruses. ZIKV is an RNA virus containing 10,794 nucleotides encoding 3,419 amino acids. The virus was isolated for the first time from a rhesus monkey in the Zika Forest of Uganda and developed fever. In 1948, ZIKV was also isolated from *Aedes africanus* mosquitoes trapped in the same forest. From 1951 through 1981, serologic evidence of human ZIKV disease was reporting by other African countries. In 2007, an outbreak was identified on Yap Island in Micronesia.^1^

An outbreak in French Polynesia began at the end of October, 2013. Around 10,000 cases were registered, of which approximately 70 were severe cases that presented neurological (Guillain Barré Syndrome [GBS], meningoencephalitis) or autoimmune (thrombo-cytopenic purpura, leukopenia) complications.^2^ The vectors responsible for transmission were *Aedes aegypti* and *Aedes polynesiensis*. In 2014, some cases were registered in New Caledonia and in the Cook Islands.^2^

ZIKV is transmitted by mosquitoes of the genus *Aedes*, both in urban areas (*A. aegypti*) and in the wild.2 The information regarding the pathogenesis of ZIKV is scarce but mosquito-borne flaviviruses are thought to replicate initially in dendritic cells near the site of inoculation and then spread to lymph nodes and the bloodstream.^3^

ZIKV incubation period ranges from 2 to 13 days. The disease is characterized by fever, joint pain (mainly of small joints), myalgias, headache, retro-orbital pain, non-purulent conjunctivitis, and maculopapular rash. The symptoms last for 4 to 7 days and are self-limited. In most cases the infection is asymptomatic.^4^

Recent reports indicate a new particularity of ZIKV: it can be sexually transmitted, in addition to perinatal and transplacental transmission during childbirth and possibly transmission by blood transfusion.^5^

**Epidemiological situation in the Americas**

Since 2014, autochthonous circulation of ZIKV has been detected in the Americas. In February 2014, the public health authorities of Chile confirmed the first case of autochthonous transmission of ZIKV in Easter Island (Isla de Pascua). In May 2015, the public health authorities of Brazil confirmed the autochthonous transmission of ZIKV in the northeastern of the country. In October of that year, Colombian health authorities reported the first autochthonous case of the Zika virus disease in the State of Bolívar.^6^

On October 30, the Department of Health Surveillance of the Ministry of Health of Brazil was notified of 54 cases of newborns with microcephaly in several public and private hospitals in the State of Pernambuco; on November 2015, the Ministry of Health of that country “confirmed” the relationship between ZIKV and microcephaly in newborns. As of epidemiological week 1 of 2016, there were 3,530 microcephaly cases recorded, including 46 deaths, in 20 states and the Federal District. Between 2010 and 2014, an average of 163 microcephaly cases was recorded nationwide per year.^7^^,^ 8

On 13 January 2016, the Brazil Ministry of Health reported the detection of Zika virus genome, through the RT-PCR technique in four cases of congenital malformation in the state of Rio Grande do Norte. The cases corresponded to two miscarriages and two full-term newborns (37 and 42 weeks respectively) who died in the first 24 hours of life. Tissue samples from both newborns were also positive for ZIKV by immunohistochemistry.^8^

As of March 31, 2016, 33 countries and territories have reported autochthonous transmission of Zika virus in the Region of the Americas.^9^

**Epidemiological surveillance of Virus Zika Disease in Mexico**

In Mexico, the systematic collection, analysis, evaluation, and dissemination of relevant epidemiological information on the main conditions of health of the population (morbidity and mortality) and their determinants is carried out, through the National Epidemiological Surveillance System (Sistema Nacional de Vigilancia Epidemiológica, SINAVE).^10^ As for the epidemiological surveillance of ZIKV, there is an special, nominal system based on the previous experience with the surveillance of other vector-borne diseases, such as dengue and chikungunya, that complies with specific mandatory procedures across the country, including operational definitions for clinical detection of cases followed by all the institutions of the National Health System.^11^

Cases of ZIKV disease are detected when medical attention is sought in any of more than 20,000 public and private health care units. Cases meeting the operational definition of a “probable case” of ZIKV disease are reported within 24 hours of their detection at the local level up to the national level to the General Directorate of Epidemiology.^12^

In areas with no evidence of ZIKV circulation, a blood sample is taken for diagnosis by laboratory from all cases meeting the operational definition of “probable case”. Once the virus is identified in the area, blood sampling is decreased to 5% of cases, except in pregnant women, whose samples are taken in 100% of the cases. The samples are then processed by the National Reference Laboratory (Institute for Epidemiological Diagnosis and Reference, InDRE) and some of the State Public Health Laboratories that fulfill the quality standards. Cases are confirmed not more than 10 days after detection.^12^ The prevention and control actions are carried out since a probable case is identified, even with no laboratory confirmation.

To face this contingency in Mexico, besides the Surveillance System of ZIKV, several other surveillance systems, such as those for vector-borne diseases (dengue, chikungunya, and West Nile Virus), the system of Acute Flaccid Paralysis, and the System of Birth Defects have been strengthened. Also, the Ministry of Health defined an Action Plan for the whole country against ZIKV named.^12^

**** **Zika virus in Mexico, and Action Plan**

Since the detection of the first autochthonous case of disease by ZIKV in the Americas, in Easter Island, Chile, in 2014, the public health authorities of Mexico have closely followed up the development of the events. Consequently, an Action Plan against the ZIKV was designed, taking as a reference the plans against dengue and chikungunya. The Plan includes the following components:^13^ 1) Health promotion, aimed mainly to avoid the mosquito bites through the use of personal protection measures and, eliminate breeding places of mosquitoes in households;^14^ 2) Reproductive health recommendations to strengthen antenatal control and early detection of risks in women of childbearing age and pregnant women;^15^ 3) Epidemiological Surveillance: Strengthening of the epidemiological surveillance and diagnosis confirmation by laboratory; early differential diagnosis mainly with dengue and chikungunya. In Mexico there is a network of epidemiologists working at the National Epidemiological Surveillance System and a Mexican Public Health Laboratory Network, coordinated by the National Reference Laboratory (InDRE);^12^ 4) Management and care of the patient: Training of health personnel for the diagnosis, differential diagnosis, and treatment of patients, as well as clinical management, and monitoring of pregnant women diagnosed with ZIKV, mainly in areas of risk and for those women showing ZIKV like symptoms;^16^ 5) Vector control: Entomological and virological monitoring, and integrated management of vectors,^13^ 6) Communication media: Information to the population regarding ZIKV nationwide situation and, in particular in the States with the largest number of cases. The previous components are coordinated at the federal level and, replicated at State and local levels.

In Mexico, on October 21, 2015, we identified an imported case of ZIKV disease in the central state of Querétaro^17^ The individual had a history of travelling to Colombia. Subsequently, on November 25, at the same time, the first two autochthonous cases of ZIKV disease were confirmed, one in the State of Nuevo Leon and the other in the State of Chiapas (a previous year, the first autochthonous case of Chikungunya was identified also in Chiapas).^18^ The first autochthonous cases had no history of travelling abroad.

**Zika Milestones in the Region of the Americas:**
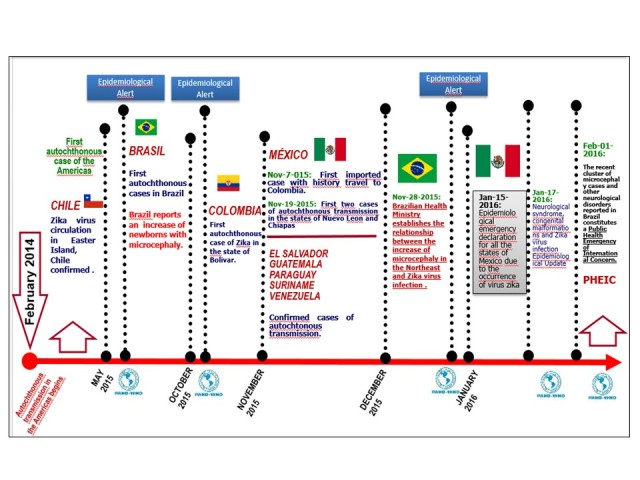
Source: World Health Organization/ Pan American Health Organization. Epidemiological Alerts and Updates. Zika Virus. Source: National Epidemiological Surveillance System (SINAVE)./ General Directorate of Epidemiology / Secretariat of Health; Epidemiological Surveillance System Zika . *Up to February 19, 2016.

At the beginning, samples were processed for dengue and chikungunya detection, but results were negative for both viruses. Blood samples of the two patients, were sent to the InDRE to evaluate the samples to ZIKV and, the results were positive.^18^

The aim of this study is to present the clinical and epidemiological characterization of 93 autochthonous cases of ZIKV disease confirmed by laboratory in Mexico, identified through the National Epidemiological Surveillance System (SINAVE) from November 25, 2015 to February 19, 2016.

## MATERIAL AND METHODS

We analyze 93 autochthonous cases of ZIKV disease identified by the Special System of Epidemiological Surveillance for Zika Virus. All autochthonous cases confirmed by laboratory since November 25, 2015 to February 19, 2016 in Mexico were included.

The Surveillance System use the next operational definitions: “Probable case”^12^ defined as any person with acute fever plus maculopapular rash and conjunctivitis (non-purulent) and, one or more of the following symptoms such as myalgia, arthralgia, headache or retro-ocular pain, and that some epidemiological association is identified. Epidemiological Association: 1) Presence of the vector *Aedes*, 2) History of visit or residence in transmission areas in the two weeks prior to the onset of symptoms, or 3) Existence of confirmed cases in the area. “Confirmed case” is consider a “probable case” laboratory with confirmation.

During the acute phase of the disease (first 5 days), samples were sent to the National Reference Laboratory or State Public Health Laboratories where viral RNA was extracted by using the QIAamp Viral RNA Mini Kit (QIAGEN, Hilden, Germany). We used real-time RT-PCR for diagnosis, using the Superscript III system (Invitrogen, Carlsbad, CA, USA) and primers and probes previously reported.^17^ We amplified a 760-bp fragment with the following primers for partial characterization of viral NS5 coding gene: ZikV9113Fwd TTYGAAGCCCTTGGATTCTT and ZikV9872Rev CYCGGCCAATCAGTTCATC. We used the QIAGEN One-Step RT-PCR Kit as follows: reverse transcription at 50°C for 30 min, followed by an activation step at 95°C for 15 min and 35 cycles of 94°C for 30 sec, 55°C for 30 sec, and 72°C for 1 min, and a final extension step at 72°C for 10 min.^19^ All cases were laboratory confirmed and reported to the Pan American Health Organization (PAHO) according to the International Health Regulations (IHR).

## RESULTS

**Sociodemographic characteristics**

The 93 cases were distributed in eight States of the country: Chiapas, 54 cases (58.1%), Oaxaca, 27 cases (29.0%); Nuevo Leon, 4 cases (4.3%); Guerrero, 3 cases (3.2%); Jalisco, Michoacan, Sinaloa,Veracruz, and Yucatan had a case each (Figure 2).

**Mexican States with authoctonous confirmed cases of Zika, Mexico, November 25, 2015 to February 19, 2016.**
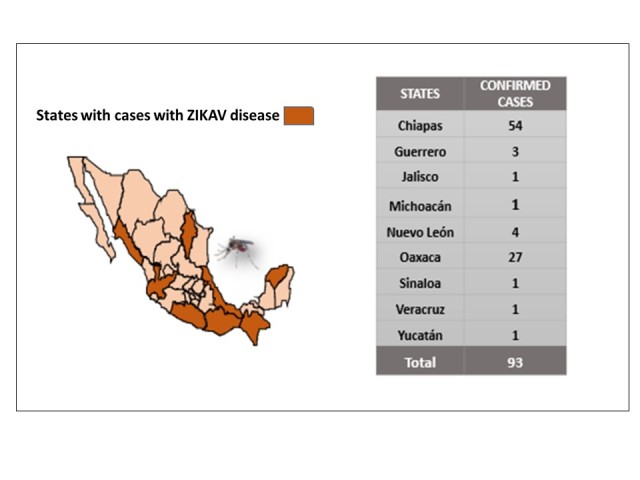
Source: National Epidemiological Surveillance System.(SINAVE)/ General Directorate of Epidemiology / Secretariat of Health; Epidemiological Surveillance System Zika . *Up to February 19, 2016.

The epidemic curve, shows that the start of the outbreak in Mexico occurred in the epidemiological week 42. An increase is observed as of the epidemiological week 51; the epidemic curve reached its peak in weeks 1 and 2 of 2016, with a subsequent decline thereafter. (Figure 3)

**Epidemic curve of autochthonous confirmed cases of Zika virus disease, Mexico November 25, 2015 to February 19, 2016.**
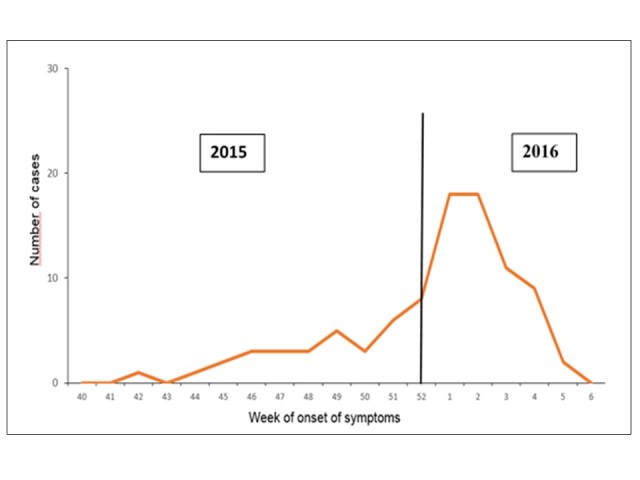
Source: National Epidemiological Surveillance System (SINAVE)./ General Directorate of Epidemiology / Secretariat of Health; Epidemiological Surveillance System Zika . *Up to February 19, 2016.

The distribution of cases according to gender was 61 (65.5%) female and 32 (34.5%) male; the mean age was 35 years (S. D. 15, range 6 – 90). By age groups, most cases corresponded to the group of 35 to 39 years old with 14 cases (15.5%), in second place the group of 25-23 years with 12 cases (12.9%), and third place with 10 cases each of the following groups 20 to 24, 40 to 44 and 45-49 years.

**Clinical characteristics**

Clinical data most frequently observed were fever (96.6%), rash (93.3%), non-purulent conjunctivitis (88.8%), headache (85.4%), and myalgia (84.3%). Other relevant clinical data were moderate/mild arthralgias (71.9%), severe polyarthralgias (20.2%), and arthritis (16.9%). (Figure 4)

**Clinical characterization of confirmed cases of Zika virus disease, Mexico November 25, 2015 to February 19, 2016.**
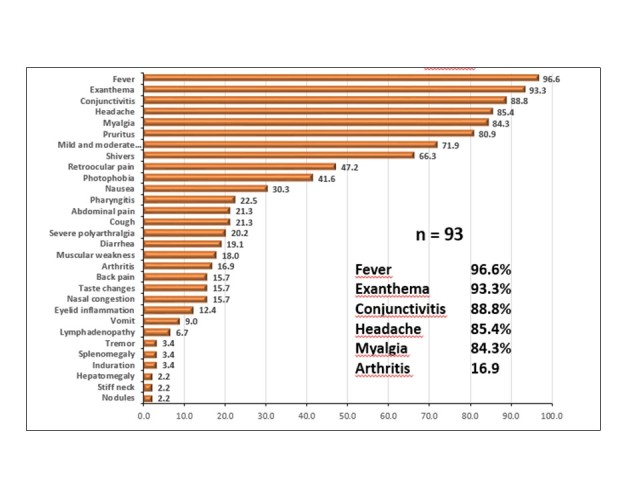
Source: National Epidemiological Surveillance System (SINAVE) / General Directorate of Epidemiology / Secretariat of Health; Epidemiological Surveillance System Zika . *Up to February 19, 2016.

Regarding to severe complications reported in the literature, such as GBS secondary to ZIKV disease, in Mexico they haven´t been reported, only were identified isoled neurological symptoms such as neck stiffness in two cases. The following clinical manifestations were identified only in one case each symptom: seizures, disorientation, and stupor.

The time elapsed between onset of symptoms and, seeking of medical care was two days on average, and the time between medical care and diagnosis confirmed by laboratory was five days on average. The prevention and control actions against ZIKAV disease initiate since the identification of suspected cases.

Among the 93 cases analyzed there are eight pregnant women, six from the State of Chiapas, one from Oaxaca, and another from Veracruz. These women were referred to receive appropriate clinical follow-up until the end of their pregnancy. One of them was diagnosed with ZIKV disease during the week 34 of pregnancy. She already had her baby, who was reported as “clinically healthy”.

The management of all patients analized was symptomatic, 91 patients were treated as outpatients and, only two required hospitalization. To date, there are no reported deaths associated with ZIKV.

## DISCUSSION

It is important to note that so far there is no standard criteria as to what cases must be reported ―suspected cases, by epidemiological association, or confirmed cases ― to the Pan American Health Organization (PAHO). This is an essential matter given that in several countries the dengue fever is endemic.^20^ and there is also the presence of Chikungunya in particular in Latin American countries9 which complicates the epidemiological situation of these diseases that share the mosquito *Aedes* as vector and whose clinical presentation may get confused in absence of well defined symptoms.^21^

The first well-documented report of human ZIKV disease was in 1964 when Simpson described his own occupationally acquired illness, it included mild headache, a maculopapular rash, fever, malaise, and back pain.^1^ Zika was characterized as a mild or inapparent dengue-like disease with fever, myalgias, eye pain, prostration, and maculopapular rash, in more than 60 years of observation.^22^ The clinical characteristics of the 93 cases confirmed by laboratory, since november, 2015 in México presents fever, rash, non-purulent conjunctivitis, headache, myalgias, and moderate/mild arthralgias, these results are consistent with the most common signs and symptoms reported previously.^23^ During the 2007 outbreak in Micronesia reported rash, fever, arthralgias, and conjunctivitis as the most common symptoms.^24^ Duffy et al. identified 49 confirmed and 59 probable cases of Zika virus disease residents on municipalities on Yap, Micronesia, fever, arthralgia, and conjunctivitis.^25^ According to WHO People with Zika virus disease usually have symptoms that can include mild fever, skin rashes, conjunctivitis, muscle and joint pain, malaise and headache. These symptoms are usually mild and last for 2-7 days.^26^ Other less frequent manifestations included headache, retro-ocular pain, edema, and vomiting.^27^

By the other hand, several studies report complications associated to ZIKAV disease, such as Guillain Barré Sindrome and neurological symptoms.^25^^,^ 28 The observation of GBS and other neurological conditions in Zika cases, represented an increase in the potential clinical severity of the disease.^22^^,^ 29

Recently on November 21, 2015, the WHO notified the presence of 739 cases of microcephaly in nine states of Northeastern Brazil^30^ the same region as the Zika virus outbreak in that country, the evidence suggest that maternal Zika virus disease is associated with adverse neonatal outcomes, most notably microcephaly.^31^^,^ 32

The ongoing epidemic confirms that Zika is predominantly a mild or asymptomatic disease, but the recent evidences regarding ZIKV and microcephaly changes the situation, and the measures to be implemented around this disease, given the impact it may have on pregnant women and, the risk involved, because the vector have a wide geographic distribution worldwide.

Moreover recent evidence regarding to other transmission mechanisms of Zika virus, without the intervention of the vector *Aedes*, such as sexual transmission^33^^,^ 34 perinatal transmission^35^ and a possibility of transmission by transfusion based on the presence of virus in asymptomatic blood donors^34^ these observations suggest that Zika virus, once introduced from an area of arboviral transmission, could lead in some cases to disease even in the absence of vector-based transmission.

Prevention and control measures are aimed at reducing vector density and exposure to mosquitoes. Basic sanitation is required to avoid the stagnation of water, for example using the strategy “Wash, Cover, Turn, and Discarded”. The actions on health education and community participation are also essential for control. The management of vector-borne diseases, such as dengue, Chikungunya, and Zika, require intersectoral actions.

## CONCLUSIONS

ZIKV epidemic poses new challenges to public health, our knowledge about this virus is incipient. In Brazil, a relationship between ZIKV and microcephaly has been mentioned since November 20157 which has not been reported in ZIKV outbreaks in other areas of the world. Several research groups are working on this topic. Guillain Barré Syndrome also has been reported as a complication of ZIKV; however, there are still many questions unanswered regarding the complications caused by this virus.

Provide information to the population, following recommendations and preventive measures, taking actions to reduce the density of the vector, and preventing mosquito bites, constitute the best tools to hold in check this Public Health Emergency of International Concern.

## Competing Interests

The authors have declared that no competing interests exist.
